# Waning Humoral Response 3 to 6 Months after Vaccination with the SARS-COV-2 BNT162b2 mRNA Vaccine in Dialysis Patients

**DOI:** 10.3390/jcm11010064

**Published:** 2021-12-23

**Authors:** Noa Berar-Yanay, Sarit Freiman, Maʹanit Shapira, Amer Saffoury, Ameer Elemy, Munir Hamze, Mohamad Elhaj, Maha Zaher, Loai Matanis, Zaher Anis Armaly

**Affiliations:** 1 Department of Nephrology, Hillel Yaffe Medical Center, Hadera 38100, Israel; 2 Laboratory Division Hillel Yaffe Medical Center, Hadera 38100, Israel; bact_lab@hy.health.gov.il (S.F.); maanit@hymc.gov.il (M.S.); 3 Rappaport Faculty of Medicine, Israel Institute of Technology, Haifa 31096, Israel; 4 Department of Victory-COVID-19, Nazareth Hospital, EMMS, Nazareth 19152, Israel; amer_saffoury@nazhosp.com (A.S.); ameer_elemy@NAZHOSP.com (A.E.); 5 Azrieli Faculty of Medicine, Bar-Ilan University, Safed 52100, Israel; 6 Department of Nephrology and hypertension, Nazareth Hospital, EMMS, Nazareth 19152, Israel; Dialysis@nazhosp.com (M.H.); fah970@gmail.com (M.E.); mahazaher@nazhsop.com (M.Z.); loai641989@gmail.com (L.M.)

**Keywords:** SAR S-COV-2, BNT162b2 mRNA vaccine, end stage renal disease, dialysis, waning

## Abstract

Background and objectives: The short-term reported antibody response to SARS-COV-2 vaccination in dialysis patients is high, with a seroconversion response rate up to 97%. Data on the long-term durability of this response are scarce. Our objective was to characterize the long-term anti-spike antibody level in dialysis patients. Design, setting, participants, and measurements: In an observational study, we measured SARS-COV-2 anti-spike antibody levels in dialysis patients who completed 2 doses of the BNT162b2 mRNA SAR S-COV-2 vaccine at 1, 3 and 6 months after the second vaccine dose. We compared the response to dialysis patients who were infected with COVD-19 and to a control group of healthcare-employees. Results: One hundred and forty-two dialysis patients who had been vaccinated (ages 64 ± 11.9 years, 61% male), 33 dialysis patients who had COVID-19 infection (ages 54 ± 14.3 years, 55% male) and 104 individuals in the control group (ages 50 ± 12.2 years, 44% male) were included. The response rate in the vaccinated dialysis patients was 94%, 78% and 73% at 1, 3 and 6 months after the second vaccine dose. In the COVID-19 infected dialysis group and in the control group, the response rate remained at 100% over 6 months. The percentage of change in antibody levels between one and 6 months was −66% in the vaccinated dialysis group, −28% in the control group (*p* < 0.001) and +48% in dialysis patients who had been infected with COVID-19 (*p* < 0.001). A non-responder status at 6 months was associated with a lower albumin level. No serious adverse events following vaccination were reported. **In conclusion:** the initially high response rate to the BNT162b2 vaccine in dialysis patients decreases rapidly. Our results indicate that an early booster (3rd) dose, at three months after the second dose, may be advised for this population to preserve the humoral immunity.

## 1. Background

Patients with ESKD are at increased risk of SARS-COV-2 (COVID-19) infection and mortality [1,2,3]. The immunogenicity to the COVID-19 vaccination in the dialysis population is lower when compared to the non-dialysis population and when compared to dialysis patients who have been infected with COVID-19. The response rate to COVID-19 vaccines in dialysis patients is reported to be between 64% and 97% [4]. While many studies report short-term antibody levels after vaccination [5,6,7,8,9,10,11,12,13], there are scarce data on the long-term antibody kinetics following COVID-19 vaccination, and clinical studies are ongoing [14]. One study reported waning antibody levels six months post vaccination [15]. Regulatory authorities, the FDA and the EMA have already recommended a booster (3rd) dose of the BNT162b2 vaccine in populations at high risk for COVID-19 infection. However, the recommended timing of the booster dose is not clear and is between 28 days [16] and at least 6 months after the second dose [17]. In an observational study, we analyzed anti-spike antibody levels over a 6-month period in dialysis patients after COVID-19 vaccination, in dialysis patients who had COVID-19 infection and in a control group of hospital employees.

## 2. Materials and Methods

### 2.1. Participants (Flow Chart)

**Group 1**: Dialysis patients who had completed 2 doses of the BNT162b2 vaccine. Patients in this group had negative anti-nucleocapsid antibody results and had not been exposed to SARS-CoV-2. Patients with breakthrough (post vaccination) infection were excluded from analysis.

**Group 2**: Dialysis patients with SARS-CoV-2 confirmed infection by positive PCR test. Only previously unvaccinated patients were included.

**Group 3**: Control group of hospital employees from all sectors who had completed 2 doses of the BNT162b2 vaccine.

**Vaccination**: All participants in Groups 1 and 3 had completed 2 doses of the BNT162b2 vaccine by 30 January 2021 (given as a 30-μg intramuscular injection, 21 days apart). Nine dialysis patients who had been infected with COVID-19 were vaccinated with one dose of BNT162b2 vaccine at least 3 months after the positive PCR result. 

### 2.2. Measurements: Anti-Spike Antibody Levels Were Measured for All Participants 

**In Group 1**: The first anti-spike antibody level was measured at one month after administration of the second dose of the vaccine, and the last measurement took place six months after administration of the second dose of the vaccine. 

In a subgroup, antibody levels were also measured 3 months after vaccination.

**In Group 2**: Anti-spike antibody levels were measured at one month and at six months after the positive SARS-COV-2 PCR result. In a subgroup, antibody levels were also measured 3 months after vaccination.

**In Group 3**: Anti-spike antibody levels were also measured one month and six months after administration of the second dose of the vaccine.

The anti-nucleocapsid antibody was measured in Groups 1 and 3 prior to each anti-spike antibody measurement. Participants with positive anti-nucleocapsid results were considered to have been exposed to SARS-COV-2 and were excluded from analysis.

### 2.3. Laboratory Methods 

Anti-spike antibodies: LIAISON^®^ SARS-CoV-2 S1/S2 IgG (DiaSorin, Sallugia, Italy), a test with high sensitivity and specificity (98% and 99%, respectively), was applied in this study. The antibodies are targeted to S1 and S2 subunits of the spike protein and correlate with neutralizing antibodies. According to the kit manufacturer, the results are presented in AU/mL, with a cutoff value for positive results (responders) at ≥15 AU/mL. The upper limit for antibody levels was 400 AU/mL. For participants with a result of >400 AU/mL, a value of 400 AU/mL was assigned.

1. COVID-19 RT-PCR: TaqPath™ COVID-19 Combo Kit, Thermo Fisher Scientific.
2. Anti-nucleocapsid antibodies-Elecsys^®^ N Anti-SARS-CoV-2, Cobas^®^, Roche Diagnostics.

### 2.4. Statistical Analysis

Descriptive statistics in terms of mean (+/− standard deviation) or median (inter quartile range, IQR) and percentiles were calculated for the whole parameters. The normal distribution of the quantitative parameters was measured by the Kolmogorov–Smirnov test. As a result of this test, ANOVA, Kruskal–Wallis and Mann–Whitney U tests were used for analysis of the difference between the three groups. (Dialysis patients after COVID-19 vaccination, dialysis patients after COVID-19 infection, and a control group).

The Wilcoxon Signed Ranks test was used for the evaluation of differences in the anti-spike antibody level between the measurements. 

A repeated measure analysis was calculated for the change in anti-spike antibody levels for the independent parameters (age, gender, diabetes, dialysis modality, dialysis vintage, albumin level and Kt/V).

SPSS version 27 was used for all statistical analysis.

*p* < 0.05 was considered significant.

*p* values for comparison between group are expressed as follows:

*p*_1_—comparison between Group 1 (post vaccination dialysis patients) and Group 2 (post infection dialysis patients).

*p*_2_—comparison between Group 1 (post vaccination dialysis patients) and Group 3 (control group).

*p*_3_—comparison between Group 2 (post infection dialysis patients) and Group 3 (control group).

Ethics:

The study was approved by the IRB, and participants signed an informed consent.

This study followed the Strengthening the Reporting of Observational Studies in Epidemiology (STROBE) reporting guideline for cohort studies.

## 3. Results

One hundred and forty-two dialysis patients who had been vaccinated (ages 64 ± 11.9 years, 61% male), 33 dialysis patients who had COVID-19 infection (ages 54 ± 14.3 years, 55% male) and 104 individuals in the control group (ages 50 ± 12.2 years, 44% male) were included (Table 1).

The response rate in the vaccinated dialysis group (% patients with a positive antibody result) was 94%, 78% and 73% at 1, 3 and 6 months after the second vaccine dose. In the COVID-19 infected dialysis group and in the control group, the response rate remained at 100% over 6 months.

### 3.1. Anti-Spike Antibody Levels over 6 Months (Median (IQR) 

**Figure 1:**
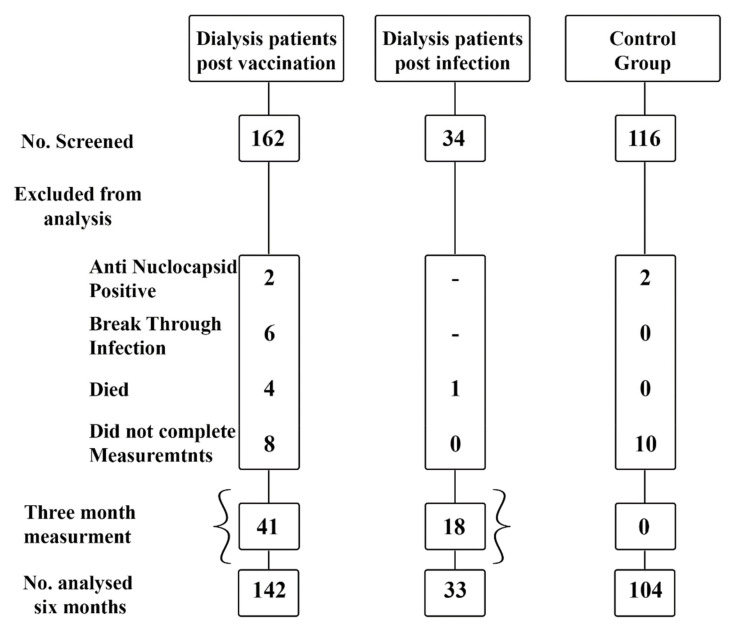
Flow Chart of the studied dialysis patients and healthy controls. The current observational study included 142 dialysis patients who completed 2 doses of the BNT162b2 mRNA SAR S-COV-2 vaccine at 1, 3 and 6 months and after the second vaccine dose were measured. We also measured SARS-COV-2 anti-spike antibody levels in dialysis patients who were infected with COVD-19 (*n* = 33) as well as a control group of healthcare-employees (*n* = 104).

One-month measurement:

Group 1: 118 AU/mL (70–157), Group 2:116 AU/mL (62–193), Group 3:186 AU/mL (144–232); *p*_1_ = 1.00; *p*_2_ < 0.001; *p*_3_ < 0.001 (Figure 1).

Three-month measurement:

Group 1: 63 AU/mL (24–100); Group 2: 203AU/mL (120–396); *p*_1_ < 0.001.

Six-month measurement:

Group 1: 33 AU/mL (14–70); Group 2: 273 AU/mL (88–400); Group 3: 133 AU/mL (93–196); *p*_1_ < 0.001, *p*_2_ < 0.001.

In Group 1, a weak positive correlation between albumin level and antibody level was found with r = 0.263; *p* = 0.012. For all other variables, age, type of dialysis, dialysis vintage, Kt/V, diabetes and gender, we did not find a correlation with antibody levels. 

### 3.2. Percentage Change in Antibody Levels over 6 Months

**Figure 2:**
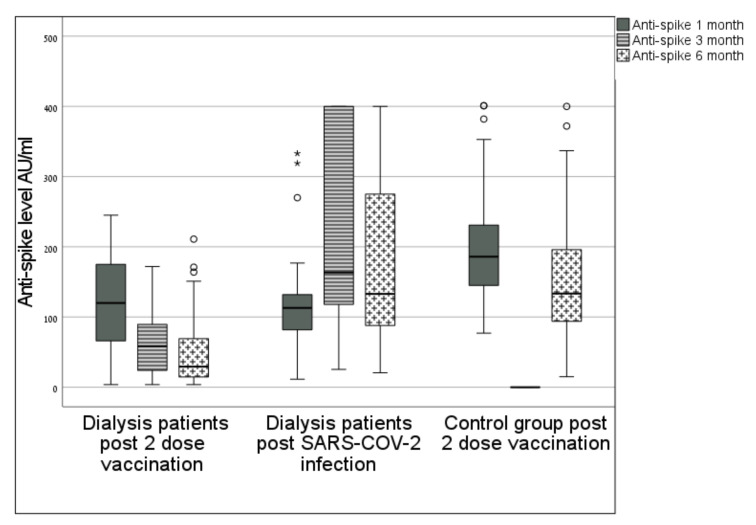
Anti-spike antibody level at 1, 3 and 6 months. Box plot diagram of anti-spike antibody levels: median, 25–75 percentiles and distribution, measured at 1, 3 and 6 months in dialysis patients post vaccination (left) and dialysis patients post infection (middle); and measured at 1 and 6 months in the control group (right); ** means significant value.

In Table 2, Figure 2, the percentage of change in antibody levels between one and six months was: −66% ((−78)–(−48)) for **Group 1**; +48% (0.4, 227) for **Group 2**; and −28% ((−38)–(−11)) for **Group 3**; *p*_1_ < 0.001, *p*_2_ < 0.001, *p*_3_ = 0.017.

In the subgroup of patients in Group 1 (*n* = 41) who had three antibody measurements, the median antibody level decreased from 118 to 63 AU/mL over the first three months, *p* < 0.001, and from 63 to 33 AU/mL over the next three months, *p* = 0.16.

### 3.3. Negative Antibody Result at 6 Months

Thirty-eight (27%) dialysis patients who had been vaccinated had a negative antibody result (<15 AU/mL) at six months while all patients who had been infected with COVID-19 and all participants in the control group remained positive for anti-spike antibodies at six months; *p*_1,2_ < 0.001 (Table 3). A lower albumin level was associated with a negative antibody result. A lower Kt/V and the presence of diabetes were numerically, but not significantly, associated with a negative antibody result. Age, gender, mode of dialysis and dialysis vintage were not associated with antibody levels.

## 4. Discussion

Our findings suggest that in dialysis patients who had been vaccinated, but not infected with COVID-19, antibody response is already decreasing three months after the second vaccine dose and continues to decline over 6 months. The initially high response rate in this group dropped from 94% to 78% after 3 months and to 73% after 6 months, while for dialysis patients who had been infected with COVID-19 and in a control group representing the general population, the antibody response rate remained at 100% over this period.

Vaccinated dialysis patients had lower anti-spike antibody levels at 1, 3 and 6 months post vaccination when compared to a control group and to dialysis patients who had the COVID-19 infection. The median level in this group was very low at 6 months.

Several factors were reported to be associated with lower response rates to vaccination in dialysis patients. The most common include older age [6,8,9,10], low serum albumin [11,12], immunosuppression [6,7,12] and diabetes [4]. We found an association between lower albumin levels and non-responder status at 6 months. We did not find a correlation between antibody levels and age, gender, mode of dialysis, dialysis vintage, dialysis adequacy or diabetes.

A waning antibody response to the BNT162b2 vaccine at 6 months in the general population and in dialysis patients has been reported [15,18]. However, our findings indicate that this decline occurs as early as 3 months post vaccination.

The reported early response rates to mRNA COVID-19 vaccines in dialysis patients are higher than response rates to other vaccines [19,20]. Based on these findings, it has been suggested that the mRNA vaccine platform may perform better than other vaccine platforms for this population [6]. However, our findings indicate that the response rate is not maintained, since there is a substantial decline in antibody levels at 3 months after vaccination. The antibody level is not equivalent to immunity [21,22]. It represents the humoral response to the vaccine while for a cellular immune response evaluation, other tests are required. Although there is no cutoff value that defines seroprotection, the anti-spike antibody level may be used as a surrogate marker [15,23] and as a guide for clinical decision making [7]. The administration of a booster vaccine is associated with an increase of antibody levels in the general population [24] and in dialysis patients [6].

Limitations of our study include measurement of humoral but not cellular response to the vaccine, such as T cells levels, and using a sample with a small group of patients. In addition, the age and gender of the control group did not match those of the patients. 

In conclusion, our findings indicate the need for an early administration of a booster vaccine dose, rather than a six month pause between the 2nd and 3rd doses, for dialysis patients who had been vaccinated and not infected with COVID-19. As data regarding the long-term durability of COVID-19 vaccines are emerging, it is possible that more specific recommendations for different patient populations will be adopted. Some questions remain to be answered: the persistence of antibody levels after the booster dose, data from other mRNA and other platform vaccines, and specific recommendations for the timing of COVID-19 vaccinations for the dialysis patient population. Furthermore, in light of the high number of “non-responders” to the COVID-19 vaccine (27% of dialysis group with <15 AU/mL at 6 months), administration of long-lasting monoclonal antibodies as prophylaxis in this group of patients may serve as protective approach for these non-responders to Pfizer vaccination. 

## Figures and Tables

**Table 1:** Patient characteristics.

	Dialysis Patients Post Vaccination (*n* = 142)	Dialysis Patients Post COVID-19 Infection (*n* = 33)	Control Group (*n* = 104)
**Type of dialysis**			
HD	115 (81%)	31 (94%)	
PD	27 (19%)	2 (6%)	
**Gender**			
Male	87 (61%)	18 (55%)	46 (44%)
Female	55 (39%)	15 (45%)	58 (56%)
Age median (25–75) Range	64 (56.7–72.3) 27–86	51 (41–62.5) 23–90	49 (40–59) 29–81
**Albumin** (g/dL) Range	3.8 (3.6–4.1) 2.6–5.0	3.9 (3.6–4.2) 2.3–5.0	
Diabetes	85 (60%)	18 (54%)	
KT/V HD, median (25–75) KT/V PD	1.42 (1.22–1.62) 2.2 (1.9–2.9)	1.55 (1.22–1.76) 1.70 (1.60–1.70)	
Dialysis vintage, years	3.4 (1.8–5.3)	3.08 (1.50–8.48)	-

HD hemodialysis; PD-peritoneal dialysis; KT/V-dialysis adequacy.

**Table 2:** Anti-spike antibody levels.

	Dialysis Patients Post Vaccination (*n* = 142)	Dialysis Patients Post COVID-19 Infection (*n* = 33)	Control Group (*n* = 104)	*p*-Value
One month Anti-spike antibody (AU/mL) median (25–75)	118 (70.3–157.0)	116 (62.3–193)	186 (144.5–232)	*p*_1_ = 1.00 *p*_2,3_ < 0.001
3-month anti-spike antibody (AU/mL) median (25–75)	63 (24–100) (*n* = 41)	203 (120–396) (*n* = 18)		*p*_1_ < 0.001
6-month anti-spike antibody (AU/mL), median (25–75)	33 (14.6–70.2)	273 (88–400)	133 (93–196)	*p*_1,2_ < 0.001
Negative anti-spike result (<15 AU/mL), no (%)	38 (27)	0	0	*p*_1,2_ < 0.001
Anti-spike level change (%) over 6 months	−66 ((−78)–(−48))	+48 (0.4, 227)	−27.7 ((−38)–(−11))	*p*_1,2_ < 0.001 *p*_3_ = 0.017
Anti-spike level change (%) 1–3 months	–51 ((−66)–(−35))	+45 ((−3)–(203))		*p* < 0.001

*p*_1_ dialysis patients post vaccination vs. post infection dialysis patients (Group 1 vs. Group 2); *p*_2_ dialysis patients post vaccination vs. control group (Group 1 vs. Group 3); *p*_3_ post infection dialysis patients vs. control group (Group 2 vs. Group 3).

**Table 3:** Anti-spike antibody status in vaccinated dialysis patients at six months.

	Anti-Spike Negative *n* = 38	Anti-Spike Positive *n* = 104	*p*-Value
Age	64.5 ± 11.4	63.5 ± 12.2	*p* = 0.67
Male Female	24 (63%) 14 (37%)	63 (61%) 41 (39%)	*p* = 0.85
HD PD	31 (82%) 7 (18%)	84 (81%) 20 (19%)	*p* = 1.00
Diabetes	25 (67%)	60 (58%)	*p* = 0.33
Albumin (gr%)	3.7 ± 0.49	3.90 ± 0.38	*p* = 0.004
Dialysis vintage (years)	3.7 ± 3.40	4.2 ± 3.55	*p* = 0.48
Kt/V HD Kt/V PD	1.37 ± 0.33 2.20 ± 0.65	1.45 ± 0.38 2.36 ± 0.57	*p* = 0.30 *p* = 0.54
One month anti-spike antibody (AU/mL) median (25–75)	41.4 (16–86)	134 (104–178)	*p* < 0.001
3-month anti-spike antibody (AU/mL) median (25–75)	8.8 (7.1–13.4)	81.5 (45.5–117)	*p* < 0.001
6-month anti-spike antibody (AU/mL) median (25–75)	8.44 (4.3–12.1)	48.4 (28.4–86.2)	*p* < 0.001

HD—hemodialysis; PD—peritoneal dialysis; Kt/V—dialysis adequacy.

## Data Availability

The study did not report any additional data.
